# Robust, Primitive, and Unsupervised Quality Estimation for Segmentation Ensembles

**DOI:** 10.3389/fnins.2021.752780

**Published:** 2021-12-30

**Authors:** Florian Kofler, Ivan Ezhov, Lucas Fidon, Carolin M. Pirkl, Johannes C. Paetzold, Egon Burian, Sarthak Pati, Malek El Husseini, Fernando Navarro, Suprosanna Shit, Jan Kirschke, Spyridon Bakas, Claus Zimmer, Benedikt Wiestler, Bjoern H. Menze

**Affiliations:** ^1^Department of Informatics, Technical University Munich, Munich, Germany; ^2^Department of Diagnostic and Interventional Neuroradiology, School of Medicine, Klinikum rechts der Isar, Technical University of Munich, Munich, Germany; ^3^TranslaTUM - Central Institute for Translational Cancer Research, Technical University of Munich, Munich, Germany; ^4^School of Biomedical Engineering & Imaging Sciences, King's College London, London, United Kingdom; ^5^Center for Biomedical Image Computing and Analytics, University of Pennsylvania, Pennsylvania, PA, United States; ^6^Department of Radiology, Perelman School of Medicine, University of Pennsylvania, Pennsylvania, PA, United States; ^7^Department of Pathology and Laboratory Medicine, Perelman School of Medicine, University of Pennsylvania, Pennsylvania, PA, United States; ^8^Department of Radio Oncology and Radiation Therapy, School of Medicine, Klinikum rechts der Isar, Technical University of Munich, Munich, Germany; ^9^Department of Quantitative Biomedicine, University of Zurich, Zurich, Switzerland

**Keywords:** quality estimation, failure prediction, anomaly detection, ensembling, fusion, OOD, CT, MR

## Abstract

A multitude of image-based machine learning segmentation and classification algorithms has recently been proposed, offering diagnostic decision support for the identification and characterization of glioma, Covid-19 and many other diseases. Even though these algorithms often outperform human experts in segmentation tasks, their limited reliability, and in particular the inability to detect failure cases, has hindered translation into clinical practice. To address this major shortcoming, we propose an unsupervised quality estimation method for segmentation ensembles. Our primitive solution examines discord in binary segmentation maps to automatically flag segmentation results that are particularly error-prone and therefore require special assessment by human readers. We validate our method both on segmentation of brain glioma in multi-modal magnetic resonance - and of lung lesions in computer tomography images. Additionally, our method provides an adaptive prioritization mechanism to maximize efficacy in use of human expert time by enabling radiologists to focus on the most difficult, yet important cases while maintaining full diagnostic autonomy. Our method offers an intuitive and reliable uncertainty estimation from segmentation ensembles and thereby closes an important gap toward successful translation of automatic segmentation into clinical routine.

## 1. Introduction

Advances in deep learning for segmentation have facilitated the automated assessment of a variety of anatomies and pathologies in medical imaging. In particular for glioma, automatic segmentation has shown great promise as a basis for objective assessment of tumor response (Kickingereder et al., 2019). In segmentation challenges such as BraTS (Menze et al., 2015), VerSe (Sekuboyina et al., 2021) and LiTS (Bilic and et al., 2019) virtually all top-performing solutions are based on ensembling. Recent efforts such as *HD-GLIO* (Kickingereder et al., 2019; Isensee et al., 2021), *GaNDLF* (Pati et al., 2021), and *BraTS Toolkit* (Kofler et al., 2020) have paved the way to apply state-of-the-art deep-learning ensembles in clinical practice. Even though algorithms often outperform human readers (Kofler et al., 2021), algorithmic reliability remains a major obstacle toward safe implementation of automated segmentation (and hence volumetry) into clinical routine (D'Amour et al., 2020). Researchers in the field of Out-of-Distribution (OOD) detection try to address this shortcoming by discovering systematic patterns within convolutional neural networks (CNN) (Schölkopf et al., 2001; Jungo et al., 2018; Mehrtash et al., 2020; Berger et al., 2021; Ruff et al., 2021). These sophisticated anomaly detection methods have the disadvantage of being limited to CNNs, often specific CNN architectures.

In contrast, we present a primitive, and therefore more applicable, solution exploiting discord in binary segmentation maps to estimate segmentation quality in an unsupervised fashion. We evaluate our method on segmentation of brain glioma in multi-modal magnetic resonance (MR)—and of lung lesions in computer tomography (CT) images. Our method allows detecting error-prone segmentation results, which require special assessment by human readers. Working only on binary segmentation maps enables our method to analyze the segmentations of human readers, classical machine learning, and modern deep learning approaches interchangeably. As segmentations are the basis for objective disease assessment as well as subsequent image analysis, our method addresses an urgent need for improving the trustworthiness of automatic segmentation methods. Furthermore, by implementing our method healthcare providers can streamline efficient use of human workforce, arguably the most persistent and major bottleneck in healthcare service worldwide (Krengli et al., 2020; Starace et al., 2020).

## 2. Methods

### 2.1. Unsupervised Quality Estimation

**Figure 1:**
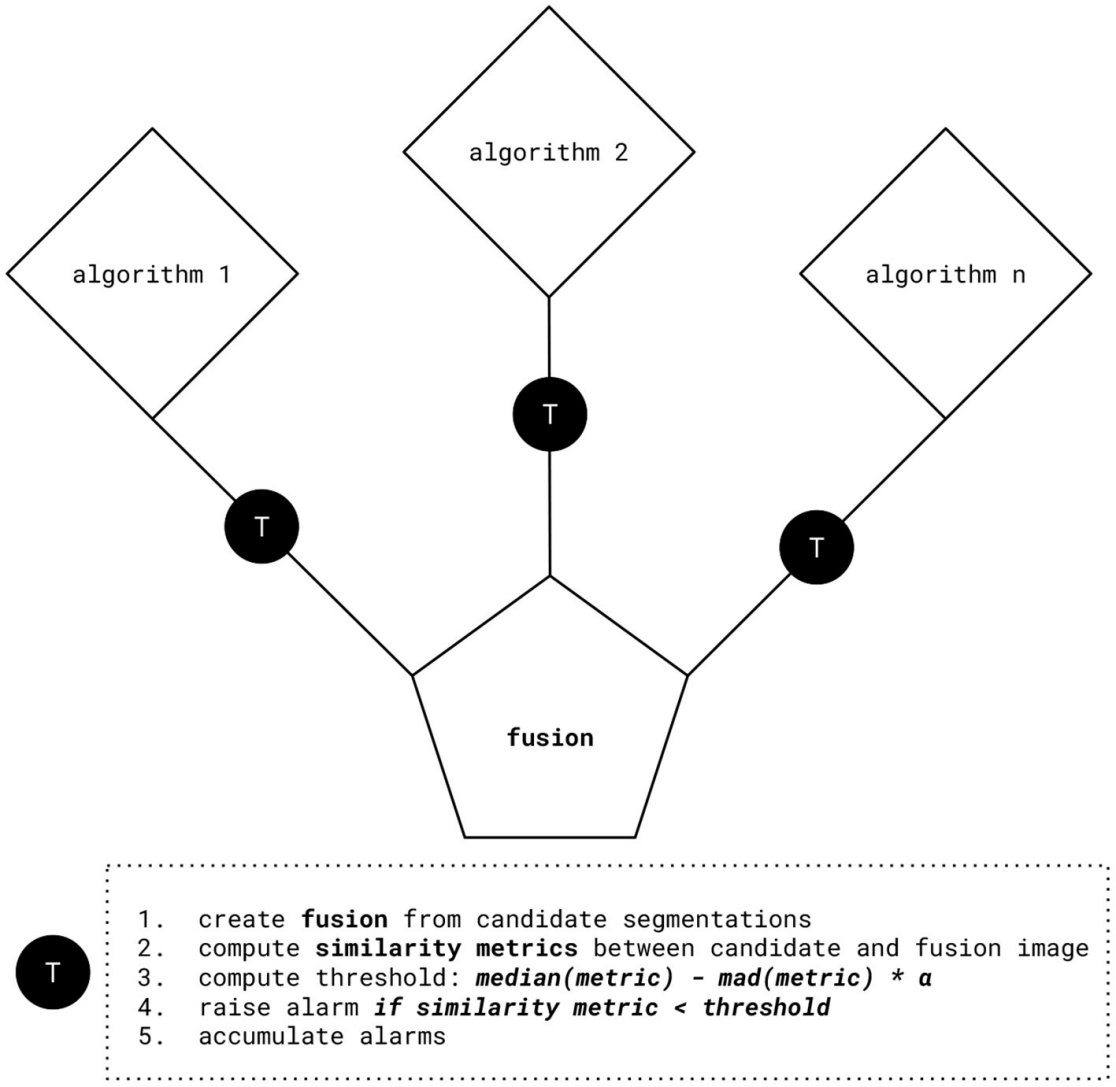
Quality estimation procedure. After computing fusion from the candidate segmentations, similarity metrics between the fused and the candidate segmentations are evaluated. Using this information, we obtain threshold values by subtracting the median absolute deviation (mad) of similarity metrics times the tunable parameter α from their median value. We set an alarm flag if the individual similarity metric is below the computed threshold, for example: *median*(*Dice*)−*mad*(*Dice*)*α.

Figure 1 depicts the quality estimation procedure. By aggregating and comparing multiple candidate segmentations, cases with large discordance, therefore a high chance of failure, can be rapidly identified. In more detail, our method consists of the following steps:

1. We obtain candidate segmentations from all methods in an ensemble, and then compute a fusion from the candidate segmentations.
2. We calculate similarity metrics between the fused segmentation result and the individual candidate segmentations.
3. We obtain the threshold for setting an alarm value by subtracting the *median absolute deviation (mad)* of the similarity metric times the tunable parameter α from its *median* value. This happens individually for each candidate image. We prefer the *median* based statistics for their better robustness toward statistical outliers. For metrics that are negatively correlated with segmentation performance, such as Hausdorff distance, we propose to use the additive inverse.
4. We set an alarm flag if the individual similarity metric is below the computed threshold. For *infinite* (or *Nan*) values, which can for instance happen for distance-based metrics such as Hausdorff distance, alarm flags are raised too.
5. Finally, we accumulate the alarm flags to obtain risk scores and therefore quality estimation for each image.

The results of this procedure are illustrated in **Figure 4**. We hypothesize that a higher count of alarm flags is associated with worse segmentation quality, here measured by lower volumetric Dice performance.

### 2.2. MR Experiment: Multi-Modal Brain Tumor Segmentation

To test the validity of our approach we use BraTS Toolkit *(btk)* (Kofler et al., 2020) to create a segmentation ensemble for brain glioma in multi-modal magnetic resonance (MR) images. Therefore, we incorporate five segmentation algorithms (Feng et al., 2019; Isensee et al., 2019; McKinley et al., 2019, 2020; Zhao et al., 2019) developed within the scope of the BraTS challenge (Menze et al., 2015; Bakas et al., 2017a,b,c, 2018). We compute alarms according to the above procedure based on Dice similarity and Hausdorff distances.

#### 2.2.1. Fusions and Segmentation Metrics

**Figure 2:**
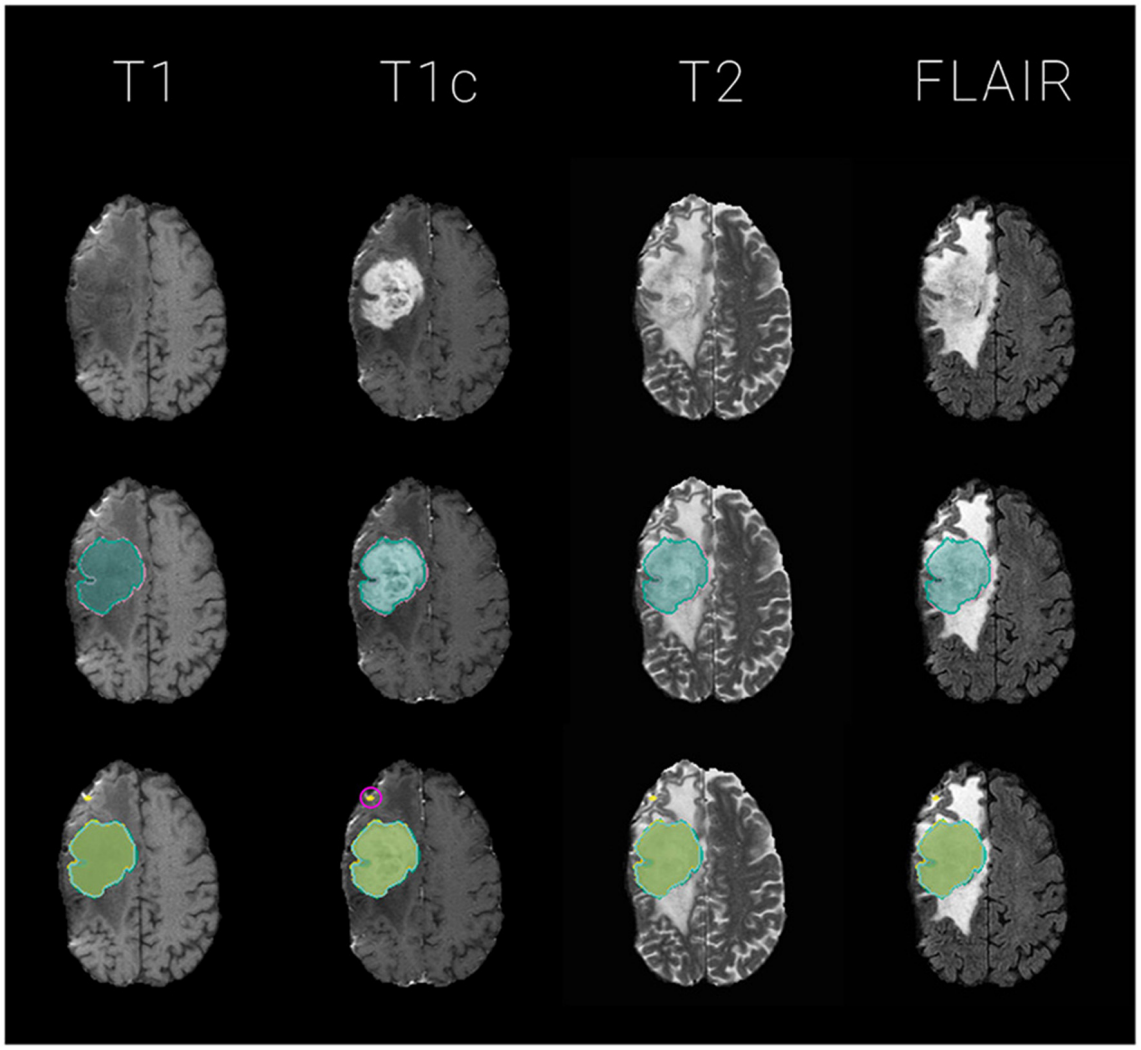
Exemplary glioma segmentation exam with multi-modal MR. Segmentations are overlayed on T1, T1c, T2, FLAIR images for the tumor's center of mass, defined by the *tumor core* (*necrosis* and *enhancing tumor*) of the ground truth label. The segmentation outlines represent the *tumor core* labels, meaning the sum of *enhancing tumor* and *necrosis* labels. **Top**: the four input images without segmentation overlay; **Middle**: ground truth segmentation (*GT*) in *reddish purple* vs. majority voting fusion (*mav*) in *bluish green*; **Bottom**: *mav* fusion in *bluish green* vs. individual segmentation algorithms in various colors. Notice the small outliers encircled in pink on the frontal lobe which probably contribute to the raise of 3 Dice - and 4 Hausdorff distance based alarms for this particular exam with a mediocre volumetric Dice similarity coefficient with the *ground truth* data of *0.66*.

We fuse the segmentations with an equally weighted majority voting using *btk* (Kofler et al., 2020) and compute segmentation quality metrics with *pymia* (Jungo et al., 2021). Figure 2 illustrates fusions and individual segmentations with an example exam.

#### 2.2.2. Data

We evaluate on a dataset of 68 cases capturing the wide diversity in glioma imaging. Our dataset consists of 15 high-grade glioma (HGG) from the publicly available Rembrandt dataset (Gusev et al., 2018), as well as another 25 HGG from TUM university hospital (MRI TUM). Furthermore, we evaluate 13 low-grade glioma (LGG) from Rembrandt and 15 from MRI TUM. Two expert radiologists generated the ground truth segmentations using *ITK-SNAP* (Yushkevich et al., 2006) and corrected each other's tumor delineations.

### 2.3. CT Experiment: COVID-19 Lung CT Lesion Segmentation

For further validation, we compose an ensemble based on the MONAI challenge baseline (MONAI CORE Team, 2020) developed for the *COVID-19 Lung CT Lesion Segmentation Challenge - 2020* (Clark et al., 2013). To segment lung lesions in computer tomography (CT) images, the code implements a 3d-Unet inspired by Falk et al. (2019). q2a1 We first train the original baseline for 500 epochs. Then we generate a small ensemble of three networks by warmstarting the training with the baseline's model weights and replacing the following parameters for the respective model for training another 500 epochs:

To obtain our first model (ADA) we swap the baseline's original Adam optimizer to *AdamW* (Loshchilov and Hutter, 2019). In a similar fashion, the second model (RAN) utilizes Ranger (Wright, 2019) to make use of Gradient Centralization (Yong et al., 2020). Our third model (AUG) adds an augmentation pipeline powered by batchgenerators (Isensee et al., 2020), torchio (Pérez-García et al., 2020), and native MONAI augmentations. In addition we switch the optimizer to stochastic gradient descent (*SGD*) with momentum (momentum = 0.95).

Our metric for training progress is the volumetric Dice coefficient. All networks are trained with an equally weighted Dice plus binary cross-entropy loss. The training is stopped once we observe no further improvements for the validation set. We conduct model selection by choosing the respective model with the best volume Dice score on the validation set. The code for the CNN trainings is publicly available via GitHub (***censored to maintain the double blind review process***).

#### 2.3.1. Fusions and Segmentation Metrics

**Figure 3:**
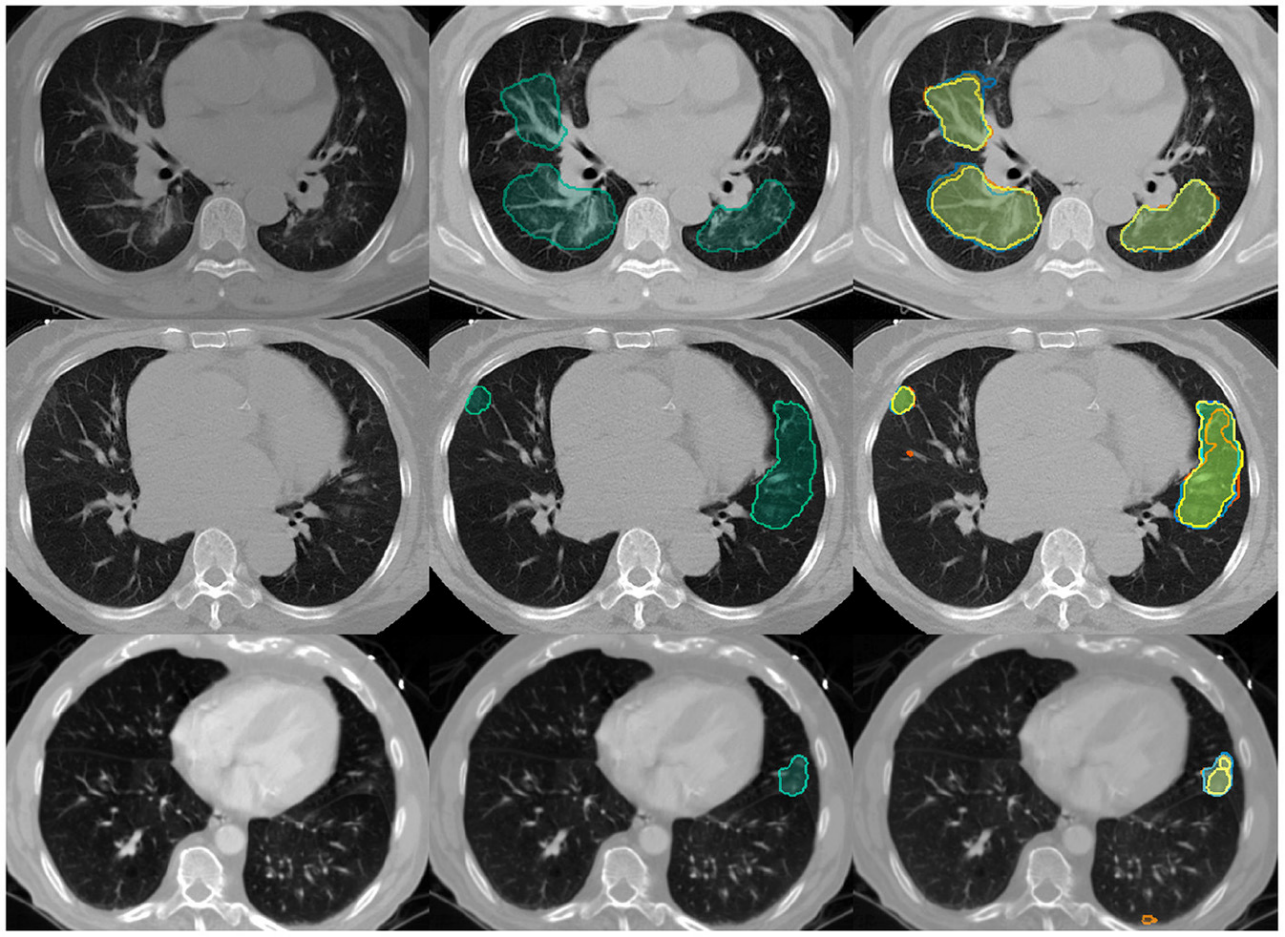
Example Covid-19 lung lesion segmentation exams with CT images. Segmentations are overlayed for the lesions' center of mass, defined by the slice with most lesion voxels: **Left**: the empty input images; **Middle**: SIMPLE segmentation fusion (simple) in *bluish green*; **Right**: SIMPLE fusion in *bluish green* vs. individual segmentation algorithms in various colors. The volumetric Dice similarity coefficients with the *ground truth* and respective alarm counts are as following: Top row: *0.81, 0*; Middle row: *0.58, 2*; Last row: *0.14, 3*.

To unify the individual outputs of our ensembles' components to a segmentation mask we choose SIMPLE (Langerak et al., 2010) fusion. SIMPLE is an iterative fusion method introduced by Langerak et al., which tends to outperform generic majority voting across various segmentation problems. An example segmentation for one exam is illustrated in Figure 3. We generate SIMPLE fusions using BraTS Toolkit (Kofler et al., 2020) and generate alarms for Dice scores calculated with *pymia* (Jungo et al., 2021). Segmentation quality metrics, in particular volumetric Dice coefficient and Hausdorff distances, for the test set are obtained through the challenge portal (COVID Challenge Team, 2021).

#### 2.3.2. Data

We run our experiments on the public dataset of the COVID-19 Lung CT Lesion Segmentation Challenge - 2020 (COVID Challenge Team, 2021), supported by the Cancer Imaging Archive (TCIA) (Clark et al., 2013).

### 2.4. Calibration of Alpha *(α*)

The α parameter can be fine-tuned to account for different optimization targets and adjusted dynamically depending on workload, e.g., in an extreme triage scenario, an alarm flag could only be raised for the strongest outliers, hence a high α should be chosen. Once the situation has been amended, α can be reset to a smaller value, resulting in a more sensitive failure prediction.

With the default value α = *0* the threshold is set to the median. Therefore, approximately half of the cases will trigger an alarm for each metric. Alternatively, alpha can be automatically adjusted to maximize the Pearson correlation coefficient with a segmentation quality metric or entropy, or combinations thereof. Tables 1, 2 illustrate how the distributions of alarm counts correlate with Dice performance and the resulting entropy in response to variations in α.

**Table 1:** Distribution of alarm counts depending on α for the MR experiment: The table illustrates the number of images classified in the individual alarm count categories *(a)* from *0* to *10*; for different values of α.

**Alpha**	**Entropy**	**r:dice**	**r:hd**	**0a**	**1a**	**2a**	**3a**	**4a**	**5a**	**6a**	**7a**	**8a**	**9a**	**10a**
−3.00	−0.00	NA	NA	0	0	0	0	0	0	0	0	0	0	68
−2.00	0.22	NA	0.04	0	0	0	0	0	0	0	0	0	4	64
−1.00	1.28	−0.27	−0.2	0	0	0	1	0	2	2	5	5	13	40
−0.75	1.80	−0.55	−0.27	0	0	1	3	4	2	10	5	4	14	25
−0.50	2.02	−0.63	−0.3	0	6	1	3	5	4	11	1	10	8	19
−0.25	2.33	−0.7	−0.38	3	5	4	5	7	4	6	7	8	7	12
−0.10	2.37	−0.73	−0.41	7	4	4	6	4	7	7	8	7	6	8
0.00	2.35	−0.76	−0.45	9	5	7	4	4	6	6	8	8	3	8
0.10	2.30	−0.77	−0.46	9	6	10	3	6	7	2	9	5	3	8
0.25	2.28	−0.77	−0.51	11	7	12	3	2	7	3	8	5	5	5
0.50	2.23	−0.78	−0.59	15	11	8	3	2	4	5	8	4	4	4
0.75	2.06	−0.73	−0.59	18	13	7	3	1	5	6	7	2	6	0
1.00	1.97	−0.72	−0.58	23	12	3	3	2	6	8	6	3	2	0
2.00	1.71	−0.66	−0.55	30	10	6	4	3	8	2	5	0	0	0
3.00	1.40	−0.65	−0.52	37	11	4	1	3	10	1	1	0	0	0

*Additionally, we depict the Pearson correlation coefficients for the Dice (r:dice) - and Hausdorff distance (r:hd) based alarm counts with volumetric Dice segmentation performance, as well as the respective alarm count distribution's entropy. The selected value for α of 0.1 is highlighted in pink The resulting computed thresholds are depicted in Table 3*.

**Table 2:** Distribution of alarm counts depending on α for the CT experiment: The table illustrates the number of images classified in the individual alarm count categories *(a)* from *0* to *3*; for different values of α.

**Alpha**	**Entropy**	**r:dice**	**0a**	**1a**	**2a**	**3a**
−3.00	−0.00	NA	0	0	0	46
−2.00	−0.00	NA	0	0	0	46
−1.00	0.58	−0.45	0	3	5	38
−0.75	0.88	−0.56	5	2	6	33
−0.50	1.19	−0.67	6	7	8	25
−0.25	1.32	−0.64	10	7	10	19
−0.10	1.36	−0.73	12	8	11	15
0.00	1.37	−0.7	13	8	14	11
0.10	1.37	−0.7	15	10	11	10
0.25	1.33	−0.62	18	9	11	8
0.50	1.20	−0.61	23	6	12	5
0.75	1.17	−0.69	25	9	8	4
1.00	1.13	−0.71	26	10	6	4
2.00	0.86	−0.67	33	8	2	3
3.00	0.66	−0.62	37	6	1	2

*Additionally, we depict the Pearson correlation coefficients for the Dice (r:dice) based alarm counts with volumetric Dice segmentation performance, as well as the respective alarm count distribution's entropy. The selected value for α of 0.1 is highlighted in pink. The resulting computed Dice similarity thresholds are as following: ADA: 0.9489; RAN: 0.9446; AUG: 0.9024*.

Note that α can also be adjusted for each segmentation target class, as well as, each of the ensemble's components, and for each similarity metric on an individual basis to fine-tune the quality estimation toward specific needs. For instance, hence the *enhancing tumor* label is of higher clinical relevance for glioma (Weller et al., 2014), one might consider setting the associated thresholds to more conservative values using a smaller *alpha*.

For simplicity, we set parameter α to *0.1* for each class, component and metric in our analysis. This results in a slightly less conservative failure prediction compared to the default.

## 3. Results

**Figure 4:**
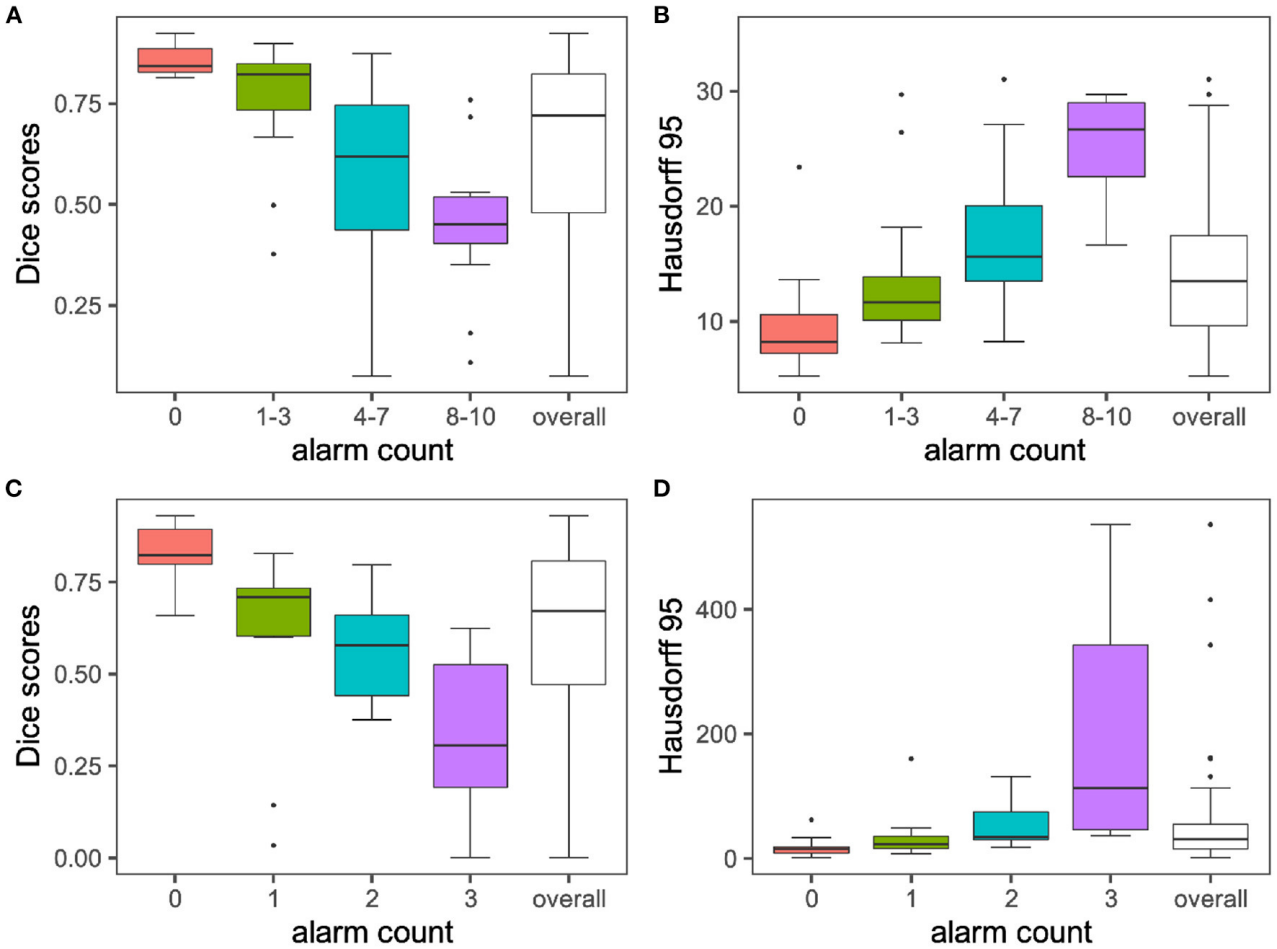
Segmentation performances vs. alarm counts. The group means are illustrated with horizontal black lines. For display purposes only the 0–95 percent quantile is displayed for Hausdorff distances on the y-axis. In line with the performance of the volumetric Dice coefficient, Hausdorff distances increase with increasing alarm count. Infinite values for Hausdorff distances, which can happen when ground truth or prediction are empty, are excluded from the plot. Subplots **(A) + (B)** illustrate findings for the MR experiment, while subplots **(C) + (D)** depict results for the CT experiment.

Our method accurately predicts the segmentation performance in both experiments and is able to capture segmentation failures. Even though our code is not optimized for speed, the computation of the fused segmentation masks, similarity metrics and resulting alarm counts is a matter of seconds. Quantitative metrics for the MR and CT experiment are summarized in Figure 4.

### 3.1. MR Experiment

Setting α to *0.1* leads to an even distribution across alarm count groups, (see Tables 1, 3). Figure 4A plots the average Dice coefficients across the tumors labels: *enhancing tumor, necrosis and edema* against the alarm count. We observe a strong negative correlation between segmentation performance and increasing alarm count: *Pearson's r* = −*0.72, p* = *3.874e-12*. This is also reflected in the Hausdorff distance, (see Figure 4B).

**Table 3:** Thresholds computed with α = 0.1 for the MR experiment per algorithm: The columns *Dice* and *Hausdorff* depict, the respective volumetric Dice and Hausdorff distance based thresholds for the alarm computation for each of the segmentation algorithms.

**Algorithm**	**Citation**	**Dice**	**Hausdorff**
micdkfz	Isensee et al., 2019	0.9055	10.2277
xfeng	Feng et al., 2019	0.9092	8.9835
scan2019	McKinley et al., 2020	0.9147	8.8292
scan	McKinley et al., 2019	0.9084	10.4850
zyx	Zhao et al., 2019	0.9293	8.4451

### 3.2. CT Experiment

Choosing an α of *0.1* leads to an even distribution across alarm count groups, (see Table 2). Figure 4C plots Dice coefficients^1^ on the challenge test set against alarm count. As for the MR experiment, we find a strong negative correlation between segmentation performance and increasing alarm count: *Pearson's r* = *-0.70, p-value* = *4.785e-08*. As observed before, this effect is mirrored by the Hausdorff distance, (see Figure 4D).

## 4. Discussion

It is important to note that, the validity of our method is closely tied to the chosen evaluation metrics' representation of segmentation performance (Kofler et al., 2021). For our experiments, we evaluate the volumetric Dice score and Hausdorff distance. Based on this fundamental assumption, we provide an unsupervised quality estimation for segmentation ensembles that does not perform any background diagnostic decisions and fully maintains the radiologists' diagnostic autonomy.

We demonstrate efficacy for two different use cases, namely multi-modal glioma segmentation in brain MR and Covid-19 lesion segmentation in lung CT images. The sensitivity of our method can be fine-tuned to specific requirements by adjusting α for ensemble components, classes, and segmentation quality metrics. Additionally, the low computational requirements make it easy to integrate into existing pipelines as computing the alarms takes only seconds and creates very little overhead.

Even though there are various efforts, such as the *BraTS algorithmic repository*^2^, to facilitate clinical translation of state-of-the-art segmentation algorithms, quality estimation mechanisms represent a currently unmet, yet important milestone on the road toward reliably deploying deep learning segmentation pipelines in clinical practice. The proposed solution can assist clinicians in navigating the plethora of exams, which have to be reviewed daily. It provides a neat prioritization mechanism, maximizing the efficient use of human expert time, by enabling focus on the most difficult, yet important cases.

It is important to note further limitations of our method. First of all, it can only be applied to model ensembles and not to single algorithms. However, as most top-performing segmentation solutions employ ensembling techniques there is a broad field of potential application. Second, the computation of alarms relies on discordance in the ensemble. If all components of the ensemble converge to predicting the same errors they cannot be detected. Notably, we did not observe such a case in our experiments, even though our CT segmentation ensemble featured only three models employing the same architecture and little variation in training parameters. As our method profits from bigger ensembles and more variations in the network training, one could argue that our experiment is probably more difficult than most real-world scenarios. Along these lines, Roy et al. (2019) activated dropout during inference and Fort et al. (2020) demonstrated that it might be enough to choose different random initialization to achieve variance in network outputs. Third, even though the default value of α, *0* and *0.1*, which we chose for demonstration purposes, performed well in our experiments, there might be segmentation problems for which α needs to be manually fine-tuned.

Future research could investigate whether α how global thresholding, instead of the proposed individual thresholding per algorithm, affects the results. It should also be explored whether the methodology can be improved by including further segmentation metrics and to which extend it generalizes to other segmentation problems.

## Data Availability Statement

Publicly available datasets were analyzed in this study. The CT data can be found here: https://covid-segmentation.grand-challenge.org/data/. The MR data will be published at: https://neuronflow.github.io/btk_evaluation/.

## Ethics Statement

Ethical review and approval was not required for the study on human participants in accordance with the local legislation and institutional requirements. Written informed consent for participation was not required for this study in accordance with the national legislation and the institutional requirements. Written informed consent was obtained from the individual(s) for the publication of any potentially identifiable images or data included in this article.

## Author Contributions

FK, IE, and LF contributed to conception and design of the study. FK, IE, CP, and JP wrote the first draft of the manuscript. All authors contributed to manuscript revision, read, and approved the submitted version.

## Funding

The annotation of the dataset was made possible through the joint work of Children's National Hospital, NVIDIA, and National Institutes of Health for the COVID-19-20 Lung CT Lesion Segmentation Grand Challenge. BM, BW, and FK are supported through the SFB 824, subproject B12. Supported by Deutsche Forschungsgemeinschaft (DFG) through TUM International Graduate School of Science and Engineering (IGSSE), GSC 81. LF, SS, and IE are supported by the Translational Brain Imaging Training Network (TRABIT) under the European Union's Horizon 2020 research & innovation program (Grant agreement ID: 765148). With the support of the Technical University of Munich - Institute for Advanced Study, funded by the German Excellence Initiative. JP and SS are supported by the Graduate School of Bioengineering, Technical University of Munich. Research reported in this publication was partly supported by the National Cancer Institute (NCI) and the National Institute of Neurological Disorders and Stroke (NINDS) of the National Institutes of Health (NIH), under award numbers NCI:U01CA242871 and NINDS:R01NS042645. The content of this publication is solely the responsibility of the authors and does not represent the official views of the NIH. Research reported in this publication was partly supported by AIME GPU cloud services.

## Conflict of Interest

The authors declare that the research was conducted in the absence of any commercial or financial relationships that could be construed as a potential conflict of interest.

## Publisher's Note

All claims expressed in this article are solely those of the authors and do not necessarily represent those of their affiliated organizations, or those of the publisher, the editors and the reviewers. Any product that may be evaluated in this article, or claim that may be made by its manufacturer, is not guaranteed or endorsed by the publisher.

## Footnotes

^1^Our basic ensemble reaches a median volumetric Dice score of *0.67*. We observe a wide performance distribution with a minimum of *0*, a maximum of *0.93* and a standard deviation of *0.25* around a mean of *0.61*, as displayed in Figure 4C. With regard to volumetric Dice coefficients mainly low-performing outliers separate our method from the top-performing methods in the challenge.

^2^ https://www.med.upenn.edu/sbia/brats2017/algorithms.html
